# Eco-Friendly Dyeing and Functional Finishing of Organic Cotton Using Optimized Oolong Tea Stems (Agricultural Waste) Through Response Surface Methodology

**DOI:** 10.3390/molecules30030509

**Published:** 2025-01-23

**Authors:** Huiya Xu, Chen Yang, Ha-young Song

**Affiliations:** 1 Department of Textile Design, Sangmyung University, Cheonan 31066, Republic of Korea; 2 Jiangxi Centre for Modern Apparel Engineering and Technology, Jiangxi Institute of Fashion Technology, Nanchang 330201, China

**Keywords:** natural dyeing, oolong tea waste, organic cotton, response surface methodology, antibacterial activity, anti-UV activity, antioxidant activity

## Abstract

As people attempt to elude the environmental issues associated with synthetic dyes, interest in natural dyes has recently increased significantly. Oolong tea stems act as a common agricultural waste yet offer advantages like high production, low cost, and a stable supply. The objectives of this research are to investigate the potential utilization of oolong tea waste, specifically tea stems, as a natural dye source for the development of organic cotton fabrics with added health benefits. In this study, using the Kubelka–Munk (K/S) value as an indicator, the dyeing process was refined through response surface methodology (RSM) by investigating the pH of the dye solution, temperature, and duration. Accordingly, it was demonstrated that the optimal effect was achieved at a pH value of 7.9, a duration of 80 min, and a temperature of 90 °C. Furthermore, under these conditions, the color fastness and functional performance of dyed organic cotton were compared with and without chitosan as a mordant. The results showed that the organic cotton dyed with oolong tea stem extract not only had a good color fastness grade but also presented excellent antibacterial properties, ultraviolet protection properties, and oxidation resistance. Especially with the assistance of chitosan, the dyed fabric achieved excellent performance above grade 4 in all color fastness test items; moreover, its antibacterial activity against Staphylococcus aureus reached more than 90%, the ultraviolet protection coefficient reached 25.3, and the antioxidant activity exceeded 90%. Consequently, considering environmental concerns, natural dyes extracted from discarded oolong tea stems are promising substitutes for synthetic dyes in the textile sector.

## 1. Introduction

Dyeing, a crucial step in textile production, enhances the fashion value of fabrics through coloring or printing [1]. Based on the sources, dyes are able to be divided into two types: natural dyes and synthetic dyes. Synthetic dyes offer advantages like great staining effects, low costs, and fitness for large-scale production [2]. However, their production and application can lead to environmental pollution [3,4,5]. Furthermore, garments made from fabrics dyed with synthetic dyes may cause chronic diseases through contact with human skin [6]. With the increasing global concern for environmental issues, the use of synthetic dye chemicals has been restricted since the early 2000s [7,8]. In contrast, natural dyes more secure for the environment and human health have gained increased attention and research interest [8,9]. These dyes encompass only organic compounds originating from renewable resources (different parts of plants and animals, some mineral compounds, and microorganisms) and are generally considered eco-friendly, as they are non-tumorigenic, non-toxic, and degradable. Especially natural dyes of plant origin have important applications in the pharmaceutical, medical, cosmetic, food, and textile industries [10]. Nonetheless, the yield and application of natural dyes draw environmental concerns, since they might originate from endangered, rare, or at-risk species [11]. Additionally, the lack of standardized raw materials for mass production, high costs, and low color yield are significant limitations that seriously restrict the industrial application of such dyes [12,13].

To address the issues described above, a growing number of researchers are focusing their efforts on agricultural waste. Agricultural waste is defined as the residues produced during the cultivation of economically valuable vegetables, fruits, crops, poultry, meat, and dairy products [13]. These agricultural waste materials are either burned or employed for non-economic purposes, resulting not only in resource waste but also in the emission of greenhouse gases, leading to adverse environmental consequences [14,15]. As a result, researchers are dedicated to investigating the reuse of agricultural waste through various innovative scientific approaches. In the textile sector, the production of natural dyes using agricultural waste has the potential to reduce both the quantity of agricultural waste and the environmental issues caused by its disposal while also providing additional income for farmers and processing industries [13]. Consequently, this area holds significant research value and market potential.

Tea (*Camellia sinensis*), as a consumed beverage, ranks second behind water worldwide [16]. In China, the most common types of tea can be categorized into six classes: green tea, black tea (commonly known as “red tea” in China), oolong tea, dark tea, white tea, and yellow tea (Table 1). These categories of tea are classified based on their degree of fermentation [17]. Specifically, green tea is classified as unfermented, while yellow tea and white tea are considered slightly fermented. Oolong tea is characterized as semi-fermented, whereas black tea is fully fermented, and dark tea is post-fermented. In terms of production volume, green tea has the highest output, followed by dark tea, black tea, and oolong tea, with white tea and yellow tea exhibiting comparatively lower production levels. Notably, the production of all types of tea has shown a consistent upward trend over the years [17,18]. Tea is a key player in human health due to its rich content of various active and functional molecules [19]. As the largest country in tea cultivation, production, and consumption [17], China produces vast quantities of waste from tea processing and consumption, including tea stems [20,21]. Oolong tea stems, occupying roughly 30% of the gross weight of raw tea, are one of the most common types of tea waste. Estimates suggest that China may produce over 100,000 tons of oolong tea stems annually [22]. These stems, unsuitable for making tea, are often discarded, leading to resource wastage. Utilizing tea stems for fabric-dyeing purposes offers an opportunity to enhance their economic value and promote sustainable development by fostering ecological balance.

As the most significant plant fiber, cotton is extensively used, particularly in the clothing industry. However, globally, some pesticide chemicals used by conventional cotton farmers are considered harmful by the World Health Organization (WHO). These pesticides have detrimental effects on soil quality, crop yield, and groundwater resources [23]. In contrast, organic cotton fibers are crucial raw materials for enhancing textile sustainability. They are cultivated naturally with no industrial agricultural chemicals like fertilizers and insecticides or transgenic technologies. In order to attain an organic cotton certification, conventional chemicals, encompassing growth regulators, insecticides, herbicides, and defoliants, must be banned for at least three years. This approach supports the ecological environment while protecting human health [24].

Numerous researchers are currently conducting studies in the field of natural dyeing, with a significant focus on tea dyeing. However, most of these investigations center around green tea, while oolong tea has received comparatively less attention. Nattaya Vuthiganond et al. extracted dyes from oolong tea leaves and performed dyeing experiments on silk, demonstrating that the abundant presence of tea polyphenols in the leaves not only resulted in excellent color performance but also imparted ultraviolet (UV) resistance to the silk after dyeing [25]. Based on in situ aggregation, Yanfei Ren developed an innovative mechanism for coloring cotton fabrics using oolong tea extracts, resulting in gently colored textiles with good color fastness and satisfying antibacterial activity against *Escherichia coli* and *Staphylococcus aureus* [26]. While the aforementioned studies utilized oolong tea leaves as experimental materials, there is a paucity of research on dyeing using oolong tea waste products. Yanfei Ren et al. investigated the effects of the dye bath pH on color fastness, features, antibacterial properties, and UV protection performance when dyeing wool fabrics with oolong tea dusts. The results indicated that dye bath pH significantly influenced the apparent color and intensity, with a maximum intensity observed at pH 5.5. Furthermore, tea endowed wool fabrics with great traits of UV protection and antibacterial activity; however, the antibacterial activity declined in acidic or alkaline conditions [27]. Li Yang et al. compared the dyeing effects of oolong tea stems and leaves, demonstrating no significant discrepancies between them for fabrics other than silk chiffon and Tencel [28].

The aforementioned studies investigated the dyeing effects and functional properties of oolong tea extracts from diverse perspectives. In our opinion, no study has investigated the dyeing and functional verification of organic cotton fibers using tea stems as raw materials. Notably, Jixian Gong et al. compared the dyeing effects of tea polyphenols on cellulose fibers (cotton) and protein fibers (wool, silk), demonstrating superior dyeing performance on wool and silk, while cellulose fibers exhibited a relatively low dye uptake rate, resulting in lighter colors [29]. This difference arises from the distinct binding patterns of tea pigments with different fabric types. Protein fabrics involve electrostatic and intermolecular forces, whereas cellulose fibers primarily involve intermolecular hydrogen bonds and van der Waals forces [29], which are weak intermolecular forces with significantly lower bond energies compared with those of ionic bonds [30]. Consequently, it is imperative to enhance the dyeing effect of tea pigments on cellulose fibers through various methods to facilitate their broader application. Existing research primarily focuses on improving the dyeing effect of tea pigments on cellulose fibers using various mordants or fabric modifications, with limited exploration of the dyeing conditions themselves. Sukemi et al. investigated an approach to coloring cotton fibers using natural dyes from green tea waste leaves. However, thus far, no research has examined the dyeing conditions for oolong tea stems on organic cotton [31]. Therefore, this study aims to determine optimal dyeing conditions through experiments, providing new insights into the application of dyes extracted from oolong tea stems to cellulose fibers. Utilizing oolong tea stems for dyeing not only mitigates environmental pollution and resource waste associated with tea waste disposal but also offers a cost-effective alternative to tea leaves, with oolong tea stems being only one percent of the price [32]. Thus, if oolong tea stems can be successfully employed as a dye source, it will significantly reduce the economic cost of natural dyeing, offering both environmental and economic benefits.

This study analyzed the phytochemical composition of tea stem extracts as natural dyes when dyeing organic cotton fabrics. The study employed response surface methodology (RSM) to identify optimal process conditions for achieving high color intensity. Furthermore, the study compared dyeing performance, antibacterial properties, UV resistance, and antioxidant properties under chitosan-mediated and non-chitosan-mediated conditions. The reason for choosing chitosan as mordant is that it is a natural polysaccharide with good biocompatibility and no toxicity. According to the existing literature, chitosan can significantly improve the dye absorption rate, color depth, and color fastness grade during the dyeing process, thereby achieving a uniform and lasting dyeing effect, showing great potential to replace traditional metal mordants [33,34,35,36]. Therefore, the natural mordant chitosan was selected in this experiment, so as to reduce the harm to the environment as much as possible in the whole dyeing process. By investigating the viability of using oolong tea waste, specifically tea stems, as a natural textile dye, this research offers novel perspectives and frameworks for the repurposing of agricultural waste.

## 2. Experimental Section

The experimental process was divided into two distinct phases. In the first phase, our research employed an RSM design to determine the optimal conditions for applying oolong tea stem extracts to organic cotton fibers. In the second phase, the color fastness grade, antibacterial performance, UV resistance, and resistance to oxidation of the dyed fabric were rated in optimal conditions. These characteristics were then compared with those obtained using chitosan pre-mordanting conditions to evaluate the influence of the dyeing on the fabric’s properties.

### 2.1. Materials

Experimental materials: Woven plain organic cotton fabric (142 g/m^2^) was purchased from the Korean brand “Cottonvill”, and oolong tea stems were obtained from tea farmers in Fujian, China. The reagents used in the optimal dyeing process experiment in the first phase included sodium hydroxide and citric acid monohydrate, purchased from China’s Xilong Science Co., Ltd. (Shantou, China). In the second phase, functional testing utilized acetic acid, chitosan, and anhydrous ethanol procured from China National Pharmaceutical Group Chemical Reagent Co., Ltd. (Beijing, China) All reagents were regarded as laboratory-grade, analytical-grade reagents. Additionally, for antimicrobial testing, *Staphylococcus aureus* was employed as the bacterial strain, supplied by the China Industrial Microorganism Culture Collection Management Center (Shanghai, China).

### 2.2. Methods

#### 2.2.1. Aqueous Extraction of Dye

The collected oolong tea waste (tea stems) was rinsed with tap water for dusting and subsequently dried. After drying, the waste was ground for 1 min using a grinder to produce a dye powder. Then, the powder was placed into a beaker to facilitate dye extraction in an aqueous medium. To maximize the content of tea polyphenols, we followed the optimal process conditions for the water extraction of tea polyphenols from Tieguanyin tea stems. Specifically, the proportion of materials to liquids reached 1:34; the temperature was maintained at 87 °C constantly; and the duration reached 57 min [28,32]. According to the experimental conclusion of Ma Xiaoqiang et al., the content of tea polyphenols was highest under this process, reaching 130.72 mg/kg [32]. In addition, Li Yang et al. used this process to extract dyes from oolong tea leaves and oolong tea stems, discussing the changes and differences in the perceptual image of dyed fabrics. The results demonstrated that there is no obvious difference in the dyeing effect between the two fabrics, and they can provide a comfortable, precious, natural, and classical feeling. This suggests the effectiveness of the extraction process and the recycling value of oolong tea stems [28].

**Figure 1:**
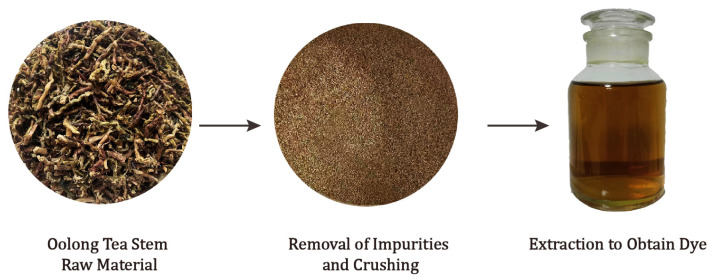
Process of dye preparation.

The extracted dye was filtrated two times to attain a dye solution, which was subsequently reserved for further dyeing processes. Figure 1 presents a diagram depicting the process of preparing the oolong tea stem dye solution.

#### 2.2.2. Phytochemical Analysis of Extracted Dye

This study employed two methods to ascertain the phytochemical composition of the aqueous extract derived from oolong tea stems, which was obtained through the water extraction method described in the preceding part.

##### UV–Visible Absorption Spectrum of Tea Stem Extract

The absorbance of the extraction solution was measured using a UV-Vis spectrophotometer (Shimadzu UV2550, Kyoto, Japan) at wavelengths of 200 nm to 700 nm. A spectral curve was plotted to determine the characteristic peaks of UV absorption.

##### Fourier Transform Infrared (FTIR) Spectroscopy of Tea Stem Extract

The infrared spectrum of the tea stem extract was obtained using an FTIR spectrometer (Thermo IS 50, Waltham, MA, USA) with the potassium bromide (KBr) precipitation method and analyzed in the wavenumber range of 400–4000 cm^−1^.

#### 2.2.3. Optimization of Dyeing Process

To determine the effective parameters influencing the staining process of oolong tea stem extract and identify the optimal conditions for achieving the highest color intensity, Design Expert software (v 10.0.7) was applied for statistical analysis. This study employed RSM and a Box–Behnken design (BBD) to refine three important operational variables of the process. Based on the principle of regression and analysis of variance, RSM is often used in various optimizations by establishing mathematical models to describe the relationship between response variables and multiple independent variables and optimizing these variables through experimental design to achieve the best response value [37]. In this study, in order to determine the best combination of experimental dyeing process parameters to obtain the best color development in organic cotton dyed with oolong tea stems, RSM was adopted. Meanwhile, the BBD is a kind of second-order response surface design based on three-level incomplete factorial design. Through a BBD, the influence of different key factors on the dyeing effect can be systematically studied in a small number of experiments [38]. The three factors in this experiment were the dye solution pH, dyeing temperature, and duration. The selection of these factors was based on prior research findings. Ren et al. concluded that the pH value of tea extract significantly impacts color intensity [27], justifying the inclusion of pH value as a factor. Furthermore, Yılmaz et al. demonstrated that different dyeing temperatures can affect the obtained colors [39], while Garg et al. showed the influence of temperature and duration on color intensify [Kubelka–Munk (K/S) value] [40]. Hence, the inclusion of dyeing duration and temperature as additional experimental factors was necessary. Before designing the experiment, the feasible range of each factor was determined through preliminary single-factor experiments, using the K/S value of the dyed fabric as the indicator. The dyeing procedure was implemented using the extracted oolong tea stem solution obtained in the previous stage.

Table 2 presents the factor levels in the response surface experiments. In light of the BBD, 17 experiments in total were executed. The quadratic regression model equation was solved, with the contour chart of the RSM analyzed to determine the optimal values for the selected parameters. After this, an analysis of variance (ANOVA) was conducted to evaluate the importance of each parameter in the prediction model. ANOVA is a statistical method applied to test whether there is a significant difference between the means of two or more samples, with a *p*-value less than 0.05 indicating statistical significance at a 95% confidence level [38,41]. In this study, the analysis of variance was employed to determine which staining factors had a significant impact on color intensity, and the reliability of the fitted equation was evaluated using the analysis of variance results. Finally, the optimal dyeing process was predicted according to the fitted regression equation model, and it was validated.

#### 2.2.4. Pre-Mordanting and Dyeing of Organic Cotton Fabrics

Prior to dyeing, the fabric samples underwent bleaching and washing to ensure complete wetting and the uniform absorption of the extract during the dyeing process. To compare the effects of direct dyeing and mordant dyeing on organic cotton, chitosan, a biological mordant agent, was employed for pre-mordanting. The chitosan solution was prepared by dissolving a specific quantity of chitosan in 2% acetic acid solution and agitating at 60 °C until fully dissolved. Subsequently, the sample fabric was mordanted at 80 °C for 60 min using a 40:1 solution-to-fabric ratio. After mordanting, the fabrics were washed using deionized water and dried in a stove at 60 °C, resulting in chitosan-modified organic cotton fabric. Under the optimal dyeing conditions determined from previous experiments, both the chitosan-pretreated and untreated fabrics were dyed using agitation. Following dyeing, excess dye materials were removed using distilled water, and samples were air dried at room temperature. Afterward, the color intensity, color fastness, and functionality of the dyed fabrics were compared and analyzed.

#### 2.2.5. Color Measurement

The K/S values and color coordinates of each dyed sample were determined using a spectrophotometer (3nh CR9, Guangzhou, China). The instrument was set as follows: 10° observer and illuminant D65. Three measurements were taken for each sample, with the mean value recorded. Moreover, the K/S values were assessed via the Kubelka–Munk equation (Equation (1)).

(1) $$\frac{K}{S}=\frac{{\left(1-R\right)}^{2}}{2R}$$

where R represents the sample reflectance at the maximal absorption wavelength (λmax = 400 nm), and K and S denote the absorption and scattering coefficients, separately.

CIELAB coordinates constitute a coordinate system employed to define color space. They are acquired by utilizing a spectrophotometer to determine the L*, a*, b*, C*, and *h°* values of dyed fabrics. In this system, L* represents the fabric’s lightness, a* indicates the red–green level, b* denotes the yellow–blue level, C* indicates saturation, and *h°* represents hue.

#### 2.2.6. Color Fastness Testing

The color fastness to rubbing, washing, perspiration, and light of the dyed organic cotton fabric was tested according to the “China Textile Color Fastness Test Standard” (GB/T 3920-2008, GB/T 3921-2008, GB/T 3922-2013, GB/T 8427-2019). These standards were formulated according to corresponding ISO international standards (ISO 105-X12:2001 [42], ISO 105-C10(2):2006 [43], ISO 105-E04:2013 [44], and ISO 105-B01:2014 [45]) [46].

#### 2.2.7. Evaluation of Fiber Surface Morphology

Employing a scanning electron microscope (ZEISS MERLIN compact, Oberkochen, Germany), this study examined the impact of oolong tea stem dyeing on the surface morphology of organic cotton fibers.

#### 2.2.8. Antimicrobial Testing

A quantitative evaluation of the antimicrobial performance of sample fabrics was conducted under AATCC 100-2004. The experiment utilized Staphylococcus aureus bacteria. The reduction percentage in bacteria (R) was used to express the antibacterial activity, as demonstrated in Equation (2):

(2) R(%)=(A−B)/A×100

Here, A represents the number of bacterial colonies obtained from the inoculated test sample after 24 h incubation, and B signifies the quantity of bacterial colonies derived from the inoculated test sample instantly after inoculation (at “0” contact time) [47].

#### 2.2.9. UV Protection Testing

As per the GB/T 18830-2009 standard [48], the UV protection factor (UPF) of fabric specimens was measured through a textile UV coefficient tester (labsphere UV-2000F, North Sutton, NH, USA) to determine the UPF, transmittance of UVA (TUVA), and transmittance of UVB (TUVB). The size of the test sample was 10 × 10 cm, with five random points tested on each fabric piece to calculate an average value. In light of the aforementioned standard, UPF can be calculated using the following formula.

(3) $$\mathrm{UPF}=\frac{\sum_{290}^{400}E_{\lambda }\times S_{\lambda }\times \Delta_{\lambda }}{\sum_{290}^{400}E_{\lambda }\times S_{\lambda }\times T_{\lambda }\times \Delta_{\lambda }}$$

Here, E_λ_ represents solar irradiance, S_λ_ is based on the erythema action spectrum of CIE, T_λ_ is the spectral transmittance of fabric, and Δ_λ_ represents the measured range of wavelengths (nm) [48].

#### 2.2.10. Antioxidant Testing

The antioxidant performance of organic cotton fibers was rated using a 2,2-diphenyl-1-picrylhydrazyl (DPPH) radical scavenging experiment. Fabric samples were mixed with an ethanol solution of DPPH and incubated in the dark for 30 min at room temperature. The absorbance was determined at 517 nm using a UV spectrophotometer, with the DPPH radical scavenging efficiency (AA) calculated using Equation (4):

(4) $$AA(\%)=\frac{\left(Ac-As\right)}{Ac}\times 100$$

where As is the sample absorbance (the residual absorbance of the solution after the reaction with the fabric sample), and Ac is the blind control absorbance (the initial absorbance of the solution without the reaction with the fabric sample).

## 3. Results and Discussion

### 3.1. Chracterization of Tea Extract

**Figure 2:**
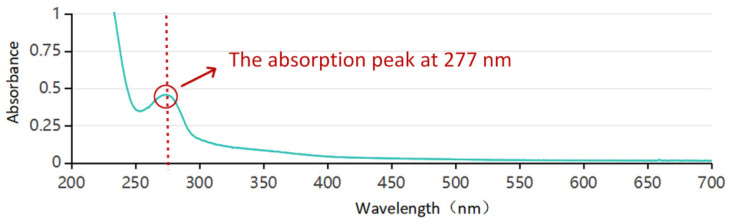
UV spectrum of aqueous extract from oolong tea stems.

Figure 2 presents the UV–visible spectrum of the aqueous extract of oolong tea stems. The maximum absorption wavelength of this extract occurred at 277 nm, corresponding to the characteristic peak (approximately 276 nm) associated with catechin-like substances, as reported in the literature [27,49]. This result confirmed the presence of catechin compounds in the oolong tea stem extract.

**Figure 3:**
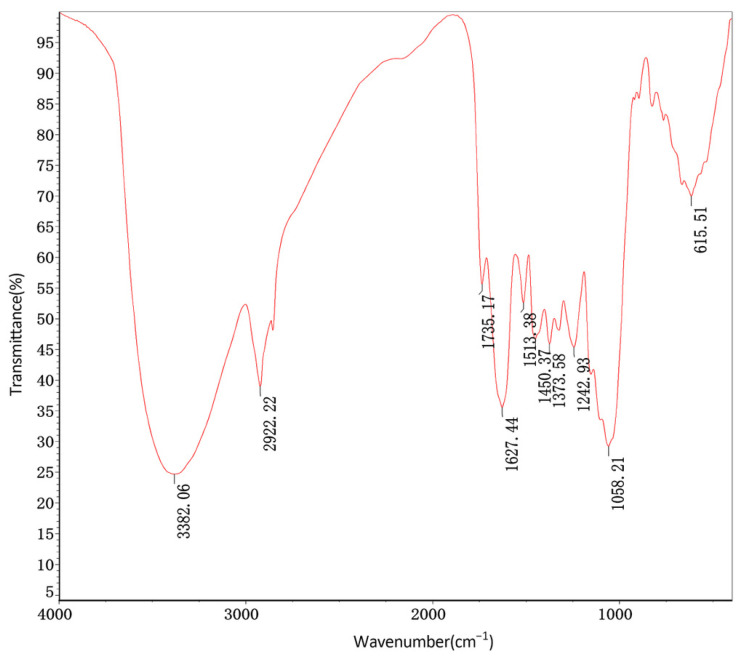
FTIR spectrum of aqueous extract from oolong tea stems.

The FTIR spectrum of the tea extract strongly supports the presence of polyphenols and catechins (Figure 3). The broad absorption band at 3382.06 cm^−1^ corresponds to the stretching vibration of –OH groups, indicating strong hydrogen bonding, which is typical of hydroxyl groups in polyphenols and catechins [50,51]. The bands at 1627.44 cm^−1^ and 1513.38 cm^−1^ are attributed to C=C stretching in aromatic rings, characteristic of the phenolic structure found in tea polyphenols and catechins [51,52]. The strong peak at 1735.17 cm^−1^ represents the C=O stretching vibration, indicative of esterified polyphenols or related structures [50,53]. The peaks at 1242.93 cm^−1^ and 1058.21 cm^−1^ correspond to C-O stretching vibrations, suggesting the presence of phenolic and ether groups commonly found in tea catechins [51,52]. Finally, the band at 615.51 cm^−1^ corresponds to aromatic C-H out-of-plane bending, consistent with aromatic ring substitutions in catechins [51,52,54]. These characteristic peaks confirmed that the main components of the extract of oolong tea stems are catechin compounds.

**Figure 4:**
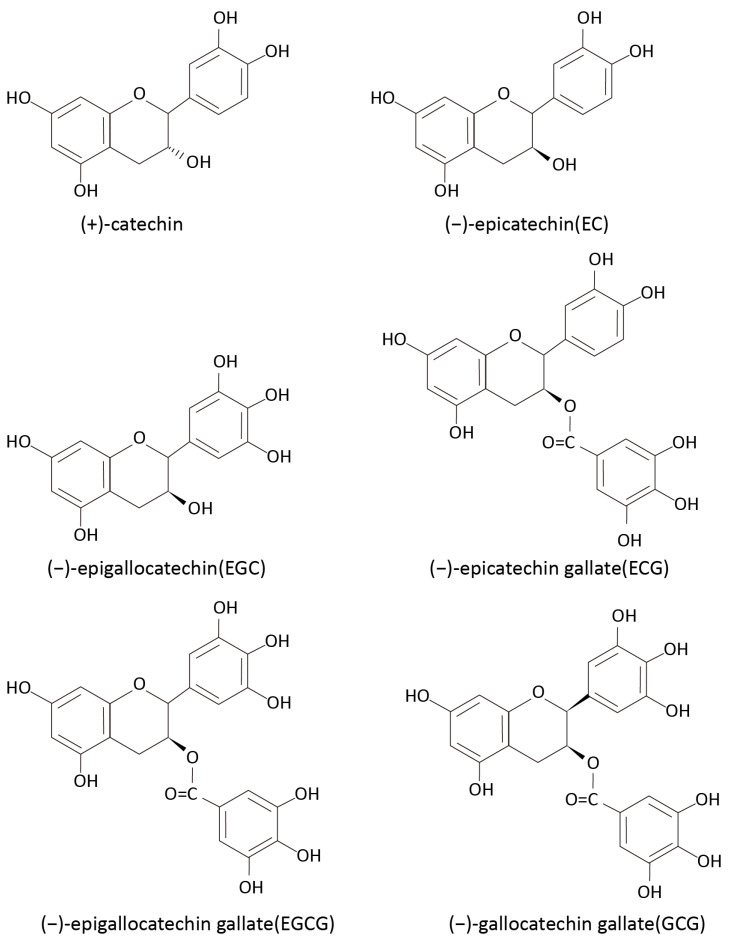
Chemical structures of (+)-catechin and (−)-epigallocatechin gallate [55].

The combined results of UV spectroscopy and FTIR spectroscopy confirmed that tea polyphenols, mainly composed of catechins, were the main components of the tea extract. Tea polyphenols, which constitute approximately 20% to 35% of tea, are rich in polyphenolic substances, with catechins as the major constituent [27], including (+)-catechin, (−)-epicatechin (EC), (−)epigallocatechin (EGC), (−)-epicatechin gallate (ECG), (−)epigallocatechin gallate (EGCG), and (−)-gallocatechin gallate (GCG), as shown in Figure 4 [55]. These polyphenols serve as precursors to tea pigments and can be converted into various types of pigments through oxidative polymerization, including theaflavins, thearubingins, and theabrownins [30]. In addition to their use as dyes, tea polyphenols offer additional health benefits and functions like antioxidant properties, antibacterial activity, and UV protection. When used as a dye in textiles, these properties can add value to fabrics [27].

### 3.2. Optimization of Dyeing Process for Organic Cotton Fabric Using Oolong Tea Stem Extract

The ideal ranges of pH, dyeing temperature, and dyeing time in the response surface optimization experiment were determined through the single-factor experiment. Furthermore, the influence of the above three factors on the color tone was examined based on the results of the single-factor experiment (Table 3). The results indicate that pH not only significantly affects color intensity but also has a significant impact on dyeing color. Under different pH conditions, there were significant differences in the color tone of dyed fabrics. Under acidic conditions, dyed fabrics tended to have a yellow hue; whereas in alkaline conditions, dyed fabrics tended to have a red hue. This can be attributed to the changes in tea polyphenols and tea pigments during the dyeing process. Oolong tea is a type of semi-fermented tea, in which some of the tea polyphenols are converted into yellow–brown theaflavins and bisflavanol during processing. During the high-temperature dyeing process, theaflavins and bisflavanol are converted into reddish–brown thearubigins by oxidative polymerization. When the dye bath was acidic, the presence of H^+^ in the solution hindered the above reaction, leading to a reduction in the production of thearubigins. Therefore, when the pH dropped to 3.5, the fabric appeared yellowish-brown. As the pH level increased, the content of thearubigins increased, causing the fabric to become more and more red [27]. On the other hand, the influence of dyeing time and temperature on the change in hue was not significant, and the dyed fabrics all showed varying degrees of brown. This means that various color changes can be achieved within the same dye system simply by adjusting the pH value.

In accordance with the BBD principles, three factors demonstrating a significant impact on the K/S value were selected: the dye solution pH, temperature, and duration. Each factor was assigned three levels, denoted as −1, 0, and 1. To measure the optimum conditions for the technical formulation, an RSM experiment consisting of 17 test points (as presented in Table 4) was conducted, with the data analyzed using statistical software.

The response variable was connected to the experimental variables by analyzing the experimental data using a second-order regression equation. This analysis adhered to a quadratic polynomial equation. Multivariate regression fitting was performed on the experimental data from Table 2 using Design Expert 10.0.7 data analysis software. The dye solution pH, dyeing temperature, and duration were indicated by A, B, and C, respectively. The K/S value was selected as the response variable for the multivariate regression fitting, yielding a second-order polynomial regression equation:

Y = 0.64 + 0.058 × A + 0.012 × B + 0.078 × C − 0.015 × AB + 0.036 × AC + 0.005 × BC − 0.18 × A^2^ + 0.046 × B^2^ + 0.007943 × C^2^

Multiple regression fitting was performed using the K/S value as the response variable. Table 5 presents the regression model coefficients and the results of significance tests.

Table 3 displays a further regression analysis of the model and its regression coefficients, confirming the statistical significance. A *p*-value less than 0.0001 denoted high significance, while the lack-of-fit term with a *p*-value of 0.7065 (*p* > 0.05) suggested no significance. This finding implies that the model can fit the experimental data well and predict corresponding values in the regression equation. Furthermore, the model’s R^2^ value was 0.9899, with an adjusted R^2^ value of 0.9769, signifying that 97.69% of the data could be interpreted by the model, demonstrating its high reliability.

The F-value acts as an important index for rating the degree of influence that various variables exert on the response value. If an F-value is higher, the model component will make a greater contribution to the response. Statistical significance is determined by the *p*-value: variables with *p* < 0.05 are considered to significantly affect the response value. In the relevant data analysis, the duration and the dye solution pH exerted extremely remarkable effects on the K/S value (*p* < 0.01), whereas dyeing temperature did not significantly affect the response (*p* > 0.05). The main factors affecting the K/S value showed the following order: duration (C) > pH value (A) > temperature (B). The quadratic interaction term AC had an extremely significant impact on the K/S value (*p* < 0.01), while AB and BC did not have a significant impact (*p* > 0.05).

**Figure 5:**
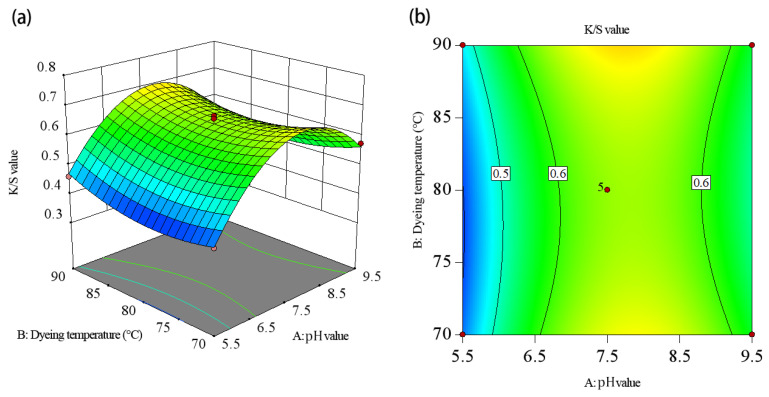
The interaction effect of the dye solution’s pH value and dyeing temperature on the K/S value: (**a**) response surface plot, (**b**) contour plot.

**Figure 6:**
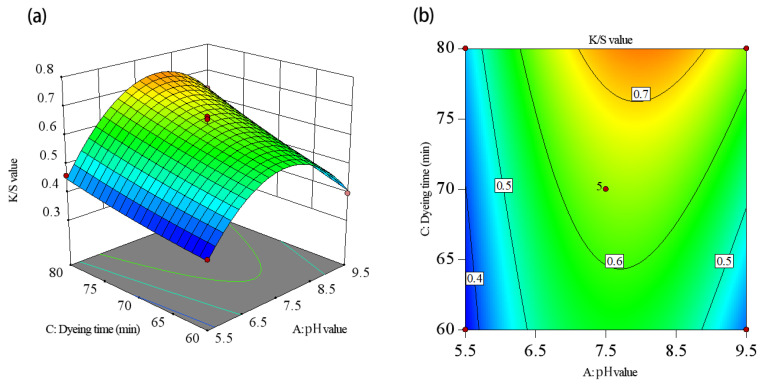
The interaction effect of the dye solution’s pH value and dyeing time on the K/S value: (**a**) response surface plot, (**b**) contour plot.

**Figure 7:**
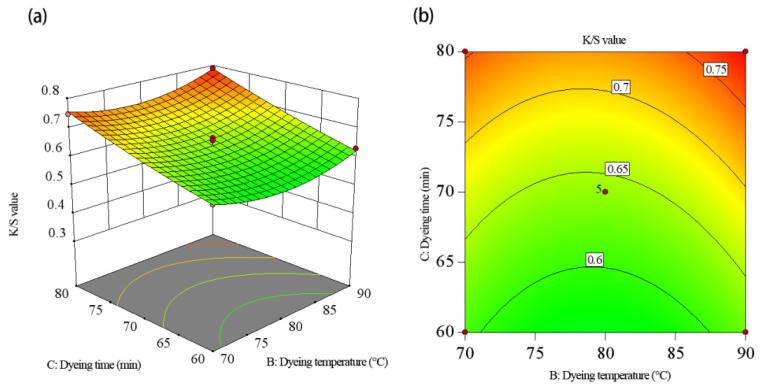
The interaction effect of dyeing temperature and dyeing time on the K/S value: (**a**) response surface plot, (**b**) contour plot.

To visualize the effects of the independent variables—the dye solution pH, duration, and temperature—response surface and contour charts were generated (Figure 5, Figure 6 and Figure 7). These charts illustrate the interactions between the variables and their impacts on the K/S value. The response surface plots show slope steepness, indicating the magnitude of each factor’s influence; steeper slopes correspond to more pronounced effects. By analyzing these plots, the interactions and optimal conditions for maximizing the K/S value could be effectively understood.

Figure 5 illustrates the effect of the interaction between the dye solution pH and temperature on the K/S value. On the AB interaction surface, as the dye solution pH increased from 5.5 to 9.5, the slope of the K/S value initially increased and then decreased. Since temperature varied between 70 and 90 °C, the K/S value exhibited a trend of slow initial increase followed by a more gradual increase. Considering only this interaction, the value achieved its maximal level when the dye solution pH reached approximately 6.5–8.5 and the temperature fell within the range of 85–90 °C. Furthermore, the effect of the dye solution pH on the K/S value was more significant than that of dyeing temperature, as the change in slope for pH was more pronounced than the change in slope for temperature. This observation is consistent with both the surface plot and the variance analysis results presented in Table 3.

Figure 6 illustrates the effect of the interaction between the dye solution pH and duration on the K/S value. The AC interaction surface unveiled that the K/S value showed a growing trend followed by a decreasing trend as the dye solution pH rose from 5.5 to 9.5. At lower dye solution pH levels, the K/S value rose with a longer dyeing duration, albeit with a limited magnitude. Conversely, at higher dye solution pH levels, the K/S value rose substantially as dyeing duration rose. This observation suggested a significant interaction effect between dye solution pH and dyeing duration. Considering this interaction effect in isolation, the K/S value attained its peak level when the dye solution pH fell within the range of 6.5–8.5 and the dyeing duration spanned 75–80 min. The surface plot corroborated the findings of the variance analysis presented in Table 3.

Figure 7 displays the interaction effect of temperature and duration on the K/S value. On the BC interaction surface, the slope of the K/S value change tended to rise as the duration varied from 60 to 80 min. The K/S value exhibited a downtrend followed by a slow increase as the temperature ranged between 70 and 90 °C. Considering only the interaction effect between these two factors, the maximal K/S value was achieved at a dyeing temperature of 85 to 90 °C and a dyeing duration of 75 to 80 min. Table 3 reveals that this result aligns with both the surface plot and the ANOVA results.

Aimed at maximizing the K/S ratio, the regression equation model predicted optimal conditions as follows: a dye liquor pH of 7.930, a dyeing temperature of 90 °C, and a duration of 80 min. Under these conditions, the quadratic fitting equation forecasts a K/S ratio of 0.796. To accommodate practical experimental constraints, the conditions were modified to a dye liquor pH value of 7.9, keeping the temperature at 90 °C and the duration at 80 min. Validation experiments conducted under these optimized parameters yielded a K/S value of 0.8279, exhibiting an error of approximately 4.01% compared to the predicted K/S value of 0.796. This deviation fell within the acceptable range of ±5%, confirming a strong agreement between predicted and experimental values. Consequently, the parameters for the dyeing process derived through the RSM are considered credible.

### 3.3. Analysis of Dyeing Effect of Oolong Tea Stem Extract on Organic Cotton

**Figure 8:**
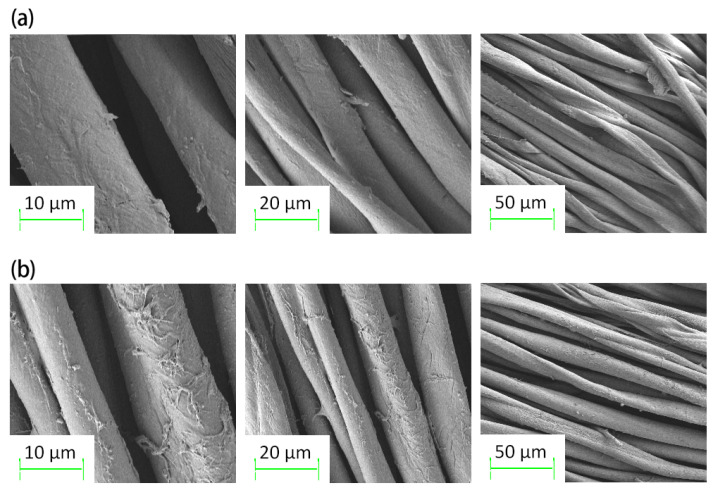
SEM views of the fabrics. (**a**) Surface of undyed organic cotton fibers, (**b**) surface of dyed organic cotton fibers.

Figure 8a presents scanning electron microscopy (SEM) images depicting the surface morphology of undyed organic cotton fibers. In comparison, Figure 8b presents SEM images of the surface morphology of the fibers following the dyeing process with oolong tea stem extract. A comparative analysis of these two images confirmed the dye adsorption on the fiber surface after staining.

**Figure 9:**
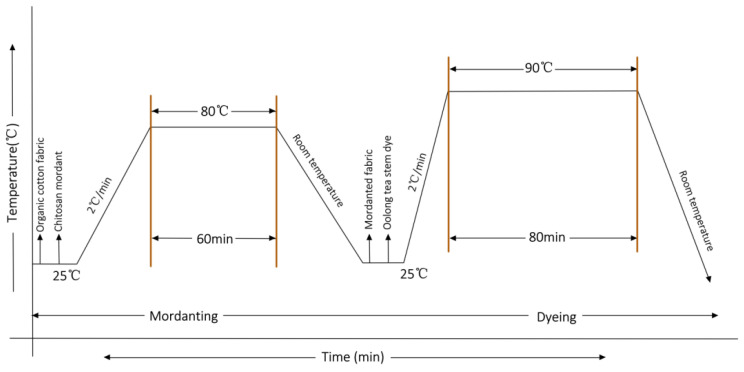
Process curve of the step-by-step dyeing procedure.

Under the optimum dyeing conditions, the overall process of the dyeing experiment is shown in Figure 9. The color parameters of undyed organic cotton, directly dyed organic cotton, and chitosan pre-mordanted dyed organic cotton were determined, as presented in Table 6. The color of dyed organic cotton fibers consistently exhibited shades in the brown series. Notably, the chitosan pre-mordanted organic cotton achieved a higher color intensity than the directly dyed organic cotton samples.

According to the color fastness standard, level 5 represents the best color fastness (with the best light fastness being level 8), while level 1 represents the worst [46]. Table 7 exhibits the color fastness (including light fastness, rubbing resistance, perspiration fastness, and washing resistance) of organic cotton fibers dyed by direct dyeing and chitosan pre-mordanting. The friction resistance test refers to the GB/T 3920-2008 standard [56]. The textile sample was rubbed against a dry friction cloth or a wet friction cloth to evaluate the degree of color transfer onto the friction cloth. The washing resistance test refers to the GB/T 3921-2008 standard [57]. In this test, a solution containing a certain proportion of non-ionic surfactant (i.e., soap) was used as the washing medium with the purpose of evaluating the performance of textiles in terms of soap washing or washing with soap and soda to simulate the cleaning process under actual household washing conditions. The perspiration resistance test refers to the GB/T 3922-2013 standard [58], where the textile specimen was sewn to a standard lining fabric and treated in an acidic or alkaline solution containing histidine. After removing the solution, the specified pressure was applied, and the color change in the sample and the staining of the lining fabric after drying were evaluated. The color fastness of the textile under simulated sunlight (D65) was assessed according to the GB/T 8427-2019 standard [59]. The data in Table 7 indicate that the rubbing fastness, washing fastness, and perspiration fastness of organic cotton fibers dyed by both methods were at least level 4, suggesting high resistance to friction, washing, and perspiration. Furthermore, while the light fastness of directly dyed organic cotton fabrics was measured at levels 3–4, the light fastness of dyed organic cotton fabrics treated with chitosan increased to level 4. This finding demonstrates that the chitosan-mediated dyeing process enhances the light fastness of organic cotton fibers dyed with oolong tea stem extract. Color fastness levels 3 and above are considered commercially acceptable [60]. The test results indicated that all evaluated items, whether subjected to direct dyeing or chitosan-mordanted dyeing, achieved a rating of level 3 or above. Consequently, it can be inferred that organic cotton fibers dyed with oolong tea stem extract show satisfying color fastness, which can be further enhanced through the application of chitosan.

**Figure 10:**
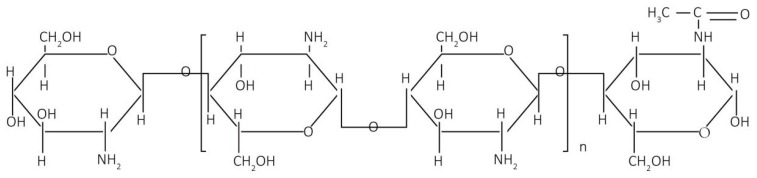
Chitosan molecular structure [64].

**Figure 11:**
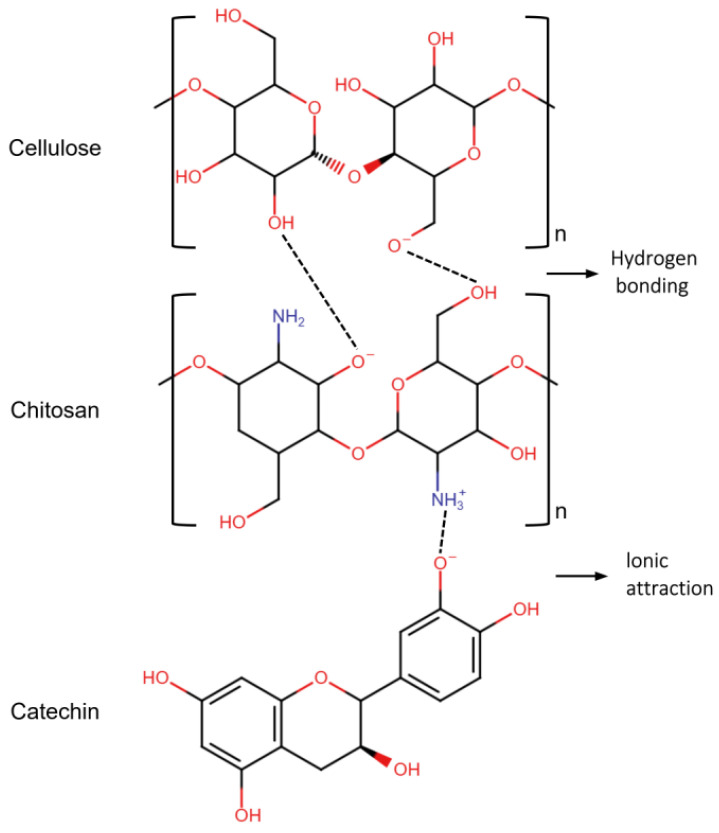
Suggested binding mechanism between fiber and catechin.

The test results for color performance and color fastness indicated that the extract of oolong tea stems exhibited an excellent dyeing effect on organic cotton. Moreover, chitosan pretreatment demonstrated good performance in enhancing color depth and improving color fastness. These outcomes can be attributed to the following primary reasons: First, chitosan introduces NH_2_ groups to the fabric structure, thus increasing the accessibility point positions for the dye molecules [61]. Integrated with van der Waals forces and hydrogen bonding, chitosan, dyes, and fibers establish a sufficient binding force to improve dye fastness [62,63]. Second, after the fiber is pretreated with chitosan, protonated amino groups (-NH_3_^+^) on chitosan molecules are deposited on the surface of the fibers. These groups can combine with negatively charged catechin coloring components through ionic attraction, leading to increased dye adsorption [55,61]. The molecular structure of chitosan is shown in Figure 10 [64], and the mechanism of action of chitosan between the fabric and the dye is shown in Figure 11. As a result, chitosan performs exceptionally well in improving fabric dye fastness and color depth.

### 3.4. Analysis of Antibacterial Effectiveness

The antibacterial properties of the dyed fabrics against *Staphylococcus aureus* were rated using the shake flask test (AATCC 100) [65]. *Staphylococcus aureus* is the most commonly studied bacterial strain and is widely present on human skin, in the nasal cavity and respiratory tract, and during household laundry processes. This bacterium is a primary cause of soft tissue and skin infections like furuncles, abscesses, and cellulitis. Moreover, *Staphylococcus aureus* is also capable of triggering severe infections, including pneumonia, bloodstream infections, and joint infections [66].

Experimentally, the results illustrated that the antibacterial effect of directly dyed organic cotton fabric was 71.89%, while the effect of dyed fabric after chitosan-mordanted dyeing exceeded 90%, as indicated in Table 8. These results indicate that the dye extracted from oolong tea stems can impart antibacterial properties to organic cotton, and chitosan can further enhance the antibacterial effect. The main reasons are threefold. First, polyphenolic compounds have received extensive attention due to their excellent antibacterial properties [67], and the extract from oolong tea stems is rich in tea polyphenols, in which the phenolic hydroxyl groups are able to form hydrogen bonds through peptide bonds with carboxyl groups and amino groups in proteins present in bacterial cell membranes and walls. Additionally, the hydrophobic benzene rings in their molecules can interact with hydrophobic groups of protein macromolecules. These multiple binding sites lead to protein denaturation, disrupting the transmembrane transport of nutrients and metabolites necessary for bacterial growth and reproduction, thereby inhibiting bacterial growth [25]. Second, after chitosan pretreatment, the surface of organic cotton is coated with protonated amino groups (-NH_3_^+^) from chitosan molecules, which can bind to negatively charged catechin coloring components through ionic attraction, increasing the dye adsorption capacity [55,61]. Thus, compared to direct dyeing of organic cotton, chitosan pre-mordanting allows the fabric to absorb more antibacterial dyes, enhancing the antibacterial effect. Third, chitosan itself possesses antibacterial properties. These antibacterial properties correlate tightly with the electrostatic interplay between charged NH_3_^+^ groups in chitosan and anionic residues within bacterial cell membranes, modifying cell osmosis and inducing osmotic instability. Furthermore, aminohydrolysis occurs in membrane-bound peptidoglycans, leading to the leakage of intracellular electrolytes and low-molecular-weight proteins like glucose dehydrogenase and nucleic acids, which hinders normal metabolism, ultimately leading to cell apoptosis [61].

### 3.5. Anti-UV Effect Analysis

The UV protection performance of organic cotton was evaluated under three conditions: undyed, directly dyed, and dyed following pretreatment with chitosan pre-mordant. The table below presents the results of this investigation.

Table 9 indicates that untreated organic cotton lacks UV protection properties. Direct dyeing with oolong tea stem dye somewhat improves the UPF value of organic cotton, but the overall effect remains moderate. However, after chitosan pretreatment and dyeing, the fabric’s UPF value surpassed 25, and the transmittance of UVA (T(UVA)) and UVB (T(UVB)) decreased significantly. Chitosan pre-mordant dyeing substantially enhanced the UV resistance performance of organic cotton fabrics compared to that of untreated or directly dyed samples. These experimental results can be ascribed to tea polyphenols [68] in oolong tea stem dye, which possess conjugated systems and functional groups such as -OH, -NH_2_, and -NH. These components inhibit high-energy UV radiation through photolytic or transformation reactions [49]. Thus, direct dyeing improves UPF compared to that of undyed fabric. However, cotton fibers have a limited capacity to adsorb dyes, resulting in moderate UV protection performance for directly dyed organic cotton fabrics. Although chitosan mordant itself does not affect the UV protection ability of mordanted fabrics [55,63], it increases the fabric’s adsorption capacity for dyes, thereby enhancing its UV protection ability.

### 3.6. Analysis of Antioxidant Effects

For the organic cotton dyed with oolong tea stem extract, the antioxidant activity is illustrated in Table 10. The data reveal that untreated fabric (control sample) exhibited a mere 2.6% antioxidant activity, while organic cotton directly dyed with the extract demonstrated an antioxidant activity exceeding 84.7%, signifying substantial antioxidative properties. Moreover, pre-mordanting with chitosan further enhanced the antioxidant activity of the dyed organic cotton, reaching levels surpassing 90%. According to the latest textile antioxidant capacity evaluation standard released by the China Cotton Textile Industry Association in 2023 [69], a sample with a free-radical scavenging rate of ≥70% can be recognized as the highest grade, indicating that it has extremely strong antioxidant capability. Regardless of whether it was colored by direct dyeing or pre-mordant dyeing with chitosan, the free-radical scavenging rate of dyed organic cotton exceeded 70%. This result indicates that the dye extracted from oolong tea stems possesses excellent antioxidant properties. This phenomenon is ascribed primarily to catechins. Free radicals are unstable molecules with unpaired electrons. These molecules try to stabilize themselves by stealing electrons from other molecules, thereby triggering chain reactions that cause cell damage and disease [70]. The phenolic hydroxyl groups in catechins can neutralize free radicals by providing one or more electrons, reducing their activity and protecting cells from oxidative damage [71]. The additional improvement in antioxidant activity observed with chitosan pre-mordanting may be ascribed to its capacity to enhance dye adsorption onto organic cotton and its interaction with active hydrogen and free radicals, transforming them into stable compounds [61]. Consequently, chitosan and oolong tea stem extract exhibit a synergistic effect on enhancing antioxidative properties.

## 4. Conclusions

### 4.1. Research Conclusions

This study utilized an extract from oolong tea stems for dyeing organic cotton fabric. Through RSM, suitable dyeing conditions were obtained: a dye solution pH of 7.9, a temperature of 90 °C, and a dyeing duration of 80 min. Based on these parameters, oolong tea stem extracts could be directly applied to organic cotton fibers, yielding a brown color in the dyed fabric. Moreover, the test results for color fastness to rubbing, washing, perspiration, and light were all above grade 3, indicating that it has good color fastness performance. Under optimal dyeing conditions, a comparison was conducted between direct dyeing and pre-mordant dyeing with chitosan as a natural mordant. The results showed that compared to direct dyeing, the use of chitosan further enhances dye absorption by fabrics and improves color intensity and color fastness. In addition, the health and functional properties of the dyed fabrics were tested. The results showed that oolong tea stem dyes impart antibacterial, antioxidant, and UV protection functions to textiles. In addition, the inclusion of chitosan as a mordant further enhances these functional performances. Test results indicated that organic cotton pretreated with chitosan and dyed with oolong tea stems exhibited over 90% antibacterial activity against *Staphylococcus aureus*, a UV protection index of 25.3, and antioxidant activity exceeding 90%. These results demonstrate the potential of oolong tea stems as a natural dye derived from common agricultural waste materials, with their coloring ability and functional characteristics further improved by the biodegradable mordant chitosan. Significantly, our research lays an excellent groundwork for the recycling of agricultural waste while providing novel insights into the design and progress of associated natural dye products.

### 4.2. Application Advantages and Challenges of Oolong Tea Stem Dyes in Textile Dyeing

Based on the results of this experiment and previous research, natural oolong tea stem dye shows tremendous potential for development and application. Its main advantages are as follows: (1) Abundant resource advantages: tea is a renewable resource, and it is the most popular beverage in many countries [17], resulting in abundant global production [72]. The extensive cultivation area ensures a stable and abundant supply of dye raw materials. (2) Advantages of environmental protection and low cost: Tea waste, including tea stems, is one of the largest sources of agricultural plant waste generated by the food industry [31], which not only causes the waste of resources but also releases greenhouse gases such as methane during the composting process, exacerbating global warming [15]. Therefore, using the large amounts of this waste as a source of raw material for natural tea dyes is feasible and has a low cost [26,27,49,73]. Using tea stems to produce natural textile dyes can not only effectively solve environmental problems in the waste treatment process [28] but also bring additional economic benefits. (3) Higher safety: Compared to the possible toxicity and irritation problems of traditional synthetic dyes, natural oolong tea stem dyes are safer and more reliable and can effectively avoid negative effects on human health when applied in textiles and other fields [25]. (4) Multiple functional characteristics: Oolong tea stems are rich in various polyphenols, which exhibit significant antibacterial, antioxidant, and anti-ultraviolet properties. These functional features not only provide better comfort and protection for the human body but also give oolong tea stems dyes great potential in the development of functional textiles. In short, in recent years, with the improvement in people’s living standards and the enhancement of environmental awareness, more and more consumers have started to focus on the safety and environmental friendliness of products [74]. Traditional synthetic dyes are gradually being questioned due to their potential safety hazards. In contrast, natural oolong tea stem dye is expected to be more widely used and developed in the future due to its rich resource advantages, environmental friendliness, safety, and functionality.

Although oolong tea stem dyes have multiple advantages in terms of economy, environmental protection, and functional performance, there are still some challenges in their application in the textile industry, mainly in the following aspects: (1) Limitations of color range: First, the color range of dyes is relatively limited, mainly consisting of different shades of brown, which may make it difficult to meet the market demand for diverse colors. (2) The stability of color is difficult to guarantee: The phytochemical components in tea stem extract are influenced by various factors (such as raw material source, climatic conditions, picking time, etc.), making it difficult to ensure product consistency [75]. The color difference between different batches or even within the same batch is a challenge in terms of color stability. (3) Complexity of technology: The processes of dye extraction and dyeing are more complex than those for synthetic dyes, requiring specific extraction and dyeing techniques, as well as usually requiring a suitable mordant to enhance color depth and fastness [29].

### 4.3. Study Limitations and Suggestions for Future Research

Due to certain objective limitations and time constraints, some related aspects were not explored deeply. Therefore, future research should further enhance and address these aspects. This study focused on the dyeing effect, antibacterial performance, UV protection characteristics, and antioxidant properties of oolong tea stem dyes on organic cotton. Future research could compare the dyeing effects on a wider range of fabrics, including linen, wool, and silk. Additionally, an exploration of other functional properties, such as mosquito repellency and flame retardancy, would reveal the application potential of oolong tea stem dyes in the textile industry. Furthermore, there are certain limitations to the color intensity and color range of the dyes extracted from oolong tea stems when applied to organic cotton. In this experiment, by using chitosan as a mordant to adjust the pH value of the dye solution, this problem was overcome to a certain extent. However, there is still room for further optimization. For example, exploring more environmentally friendly and efficient types of mordanting or dye enhancement methods, as well as how to further expand the color gamut, still needs to be further studied.

## Figures and Tables

**Table 1:** The six major types of tea in China.

Tea Type	Green Tea	White Tea	Yellow Tea	Oolong Tea	Black Tea	Dark Tea
Level of fermentation	Non-fermented tea	Slightly fermented tea (10–20% fermentation)	Slightly fermented tea (10–20% fermentation)	Semi-fermented tea (30–60% fermentation)	Fully fermented tea (80–90% fermentation)	Post-fermented tea (100% fermentation)
Liquor color	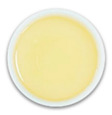	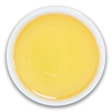	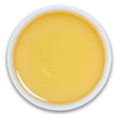	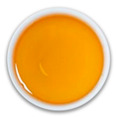	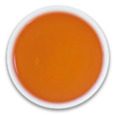	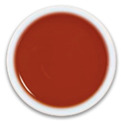

**Table 2:** Experimental factors and levels in response surface analysis.

Level	pH Value of Dye Solution (A)	Dyeing Temperature (B)/°C	Dyeing Duration (C)/min
−1	5.5	70	60
0	7.5	80	70
1	9.5	90	80

**Table 3:** Effect of pH value, dyeing temperature, and dyeing time on color tone.

**Effect of pH Value on Color Tone**					
pH value	3.5	5.5	7.5	9.5	11.5
Apparent color	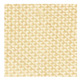	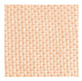	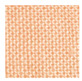	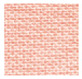	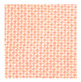
Color tone	Yellow brown	Brown	Brown	Reddish brown	Reddish brown
**Effect of Dyeing Temperature on Color Tone**					
Temperature (°C)	70	80	90	100	110
Apparent color	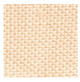	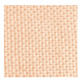	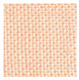	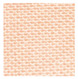	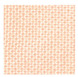
Color tone	Brown	Brown	Brown	Brown	Brown
**Effect of Dyeing Time on Color Tone**					
Apparent color	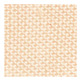	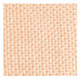	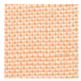	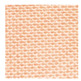	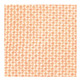
Color tone	Brown	Brown	Brown	Brown	Brown

**Table 4:** Experimental design and results of the RSM optimization for the K/S value.

Number	A—pH Value of Dye Solution	B—Dyeing Temperature (°C)	C—Dyeing Duration (min)	K/S Value
1	5.5	70	70	0.4152
2	9.5	70	70	0.5736
3	5.5	90	70	0.4604
4	9.5	90	70	0.5599
5	5.5	80	60	0.3680
6	9.5	80	60	0.3977
7	5.5	80	80	0.4601
8	9.5	80	80	0.6326
9	7.5	70	60	0.6086
10	7.5	90	60	0.6300
11	7.5	70	80	0.7466
12	7.5	90	80	0.7880
13	7.5	80	70	0.6270
14	7.5	80	70	0.6311
15	7.5	80	70	0.6177
16	7.5	80	70	0.6660
17	7.5	80	70	0.6569

**Table 5:** Table of variance for the regression model.

Source	Sum of Squares of Deviations	Freedom Degree	Average Square	F-Value	*p*-Value	Significance
Model	0.23	9	0.025	76.13	<0.0001	**
A—pH value of dye solution	0.026	1	0.026	79.25	<0.0001	**
B—dyeing temperature	0.001112	1	0.001112	3.33	0.1108	
C—dyeing duration	0.049	1	0.049	145.31	<0.0001	**
AB	0.000867	1	0.000867	2.6	0.1511	
AC	0.005098	1	0.005098	15.27	0.0058	**
BC	0.0001	1	0.0001	0.3	0.6012	
A^2^	0.14	1	0.14	422.71	<0.0001	**
B^2^	0.008762	1	0.008762	26.24	0.0014	**
C^2^	0.000266	1	0.000266	0.8	0.402	
Residual	0.002337	7	0.000334			
Lack of fit	0.00063	3	0.00021	0.49	0.7065	ns
Pure error	0.001707	4	0.000427			
Total	0.23	16				
R^2^ = 0.9899; Adj R^2^ = 0.9769; Pre R^2^ = 0.9448						

Note: ** denotes high statistical significance (*p* < 0.01); ns denotes no significance (*p* > 0.05).

**Table 6:** Color parameters of organic cotton.

Sample	Apparent Color	K/S Value	L*	a*	b*	C*	*h°*
Undyed	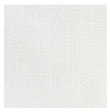	0.12	94.98	−0.68	0.25	0.72	159.8
Dyed	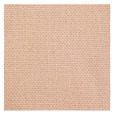	0.83	75.21	10.65	13.61	17.28	51.9
Chitosan-treated + dyed	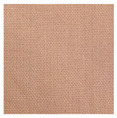	1.20	70.40	11.14	14.36	18.17	52.1

**Table 7:** Color fastness of dyed organic cottons.

Sample	Rubbing Fastness		Washing Fastness		Perspiration Fastness		Light Fastness
	Dry	Wet	Fading	Staining	Acid Staining	Alkaline Staining	
Dyed	4~5	4~5	4	4~5	4~5	4~5	3~4
Chitosan-treated + dyed	4~5	4~5	4	4~5	4~5	4~5	4

**Table 8:** Antibacterial performance of dyed organic cotton fabric samples.

Sample	Photograph	Antibacterial Rate
Dyed	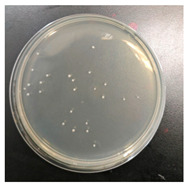	71.89%
Chitosan-treated + dyed	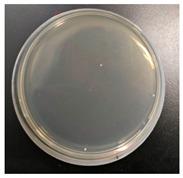	90.40%

**Table 9:** UPF of organic cotton samples.

Sample	Transmittance		UPF
	T(UVA)	T(UVB)	
Undyed (control)	14.83%	15.46%	6.22
Dyed	8.68%	6.84%	13.51
Chitosan-treated + dyed	4.11%	3.82%	25.34

**Table 10:** Antioxidant properties of dyed fabrics.

Sample	Free-Radical Scavenging Rate	Antioxidant Property
Undyed (control)	2.6%	The sample does not have antioxidant properties
Dyed	84.7%	The sample has extremely strong antioxidant properties
Chitosan-treated + dyed	90.2%	The sample has extremely strong antioxidant properties

## Data Availability

All data generated in this study are included in this paper. Further enquiries can be directed to the corresponding author.
